# Aqueous Extract of Kan-Lu-Hsiao-Tu-Tan Ameliorates Collagen-Induced Arthritis in Mice by Inhibiting Oxidative Stress and Inflammatory Responses

**DOI:** 10.3390/life10120313

**Published:** 2020-11-27

**Authors:** Chih-Chao Chiang, Yi-Rong Li, Kuei-Hung Lai, Wei-Jen Cheng, Shih-Chao Lin, Yi-Hsuan Wang, Po-Jen Chen, Sien-Hung Yang, Chi-Chien Lin, Tsong-Long Hwang

**Affiliations:** 1 Graduate Institute of Clinical Medical Sciences, College of Medicine, Chang Gung University, Taoyuan 333, Taiwan; moonlight0604@hotmail.com or misterarren@gmail.com or; 2 Puxin Fengze Chinese Medicine Clinic, Taoyuan 326, Taiwan; 3 Changhua Christian Hospital, Thoracic Medicine Research Center, Changhua 500, Taiwan; peanutsnoopyemmali@gmail.com or; 4 Research Center for Chinese Herbal Medicine, Research Center for Food and Cosmetic Safety, and Graduate Institute of Health Industry Technology, Chang Gung University of Science and Technology, Taoyuan 333, Taiwan; mos19880822@gmail.com or; 5 Graduate Institute of Pharmacognosy, College of Pharmacy, Taipei Medical University, Taipei 110, Taiwan; 6 Center for Traditional Chinese Medicine, Chang Gung Memorial Hospital, Taoyuan 333, Taiwan; 7 School of Traditional Chinese Medicine, Chang Gung University, Taoyuan 333, Taiwan; 8 National Center for Biodefense and Infectious Diseases, School of Systems Biology, George Mason University, Manassas, VA 20110, USA; slin20@gmu.edu; 9 Bachelor Degree Program in Marine Biotechnology, National Taiwan Ocean University, Keelung 202, Taiwan; 10 Graduate Institute of Natural Products, College of Medicine, Chang Gung University, Taoyuan 333, Taiwan; e0919133641@gmail.com or; 11 Department of Cosmetic Science, Providence University, Taichung 433, Taiwan; litlep@hotmail.com or; 12 Institute of Biomedical Science, College of Life Sciences, National Chung-Hsing University, 250 Guoguang Road, Taichung 402, Taiwan; 13 Department of Pharmacology, College of Medicine, Kaohsiung Medical University, Kaohsiung 807, Taiwan; 14 Department of Medical Research, China Medical University Hospital, Taichung 404, Taiwan; 15 Department of Anesthesiology, Chang Gung Memorial Hospital, Taoyuan 333, Taiwan; 16 Department of Chemical Engineering, Ming Chi University of Technology, New Taipei City 243, Taiwan

**Keywords:** Kan-Lu-Hsiao-Tu-Tan, collagen-induced arthritis, inflammation, immunity, Chinese medicine

## Abstract

Background: Kan-Lu-Hsiao-Tu-Tan (KLHTT) exhibits anti-psoriatic effects through anti-inflammatory activity in mice. However, the therapeutic effects of KLHTT on rheumatoid arthritis (RA), another significant autoimmune inflammatory disorder, have not been elucidated. Herein, we explored the anti-arthritic effects of KLHTT on collagen-induced arthritis (CIA) in mice. Methods: KLHTT was extracted by boiling water and subjected to spectroscopic analysis. Chicken collagen type II (CII) with complete Freund’s adjuvant was intradermally injected to induce CIA in DBA/1J mice. Anti-CII antibody, cytokines, malondialdehyde (MDA), and hydrogen peroxide (H_2_O_2_) were measured using ELISA, thiobarbituric acid reactive substances, and a hydrogen peroxide assay kit. Splenocyte proliferation was tested using thymidine incorporation. Th1 and Th17 cells were analyzed by flow cytometry. Results: Oral KLHTT treatment (50 and 100 mg/kg) ameliorated mouse CIA by decreasing the levels of interleukin (IL)-1β, IL-6, IL-17A, and tumour necrosis factor-α in the paw homogenates and serum. KLHTT also suppressed anti-CII antibody formation, splenocyte proliferation, and splenic Th1 and Th17 cell numbers. Additionally, KLHTT showed antioxidant activity by reducing the concentrations of MDA and H_2_O_2_ in paw tissues. Conclusions: The therapeutic effects of KLHTT in CIA mice were through regulating oxidative stress and inflammatory responses. Our results suggest that KLHTT has potential to treat RA.

## 1. Introduction

Rheumatoid arthritis (RA) is an autoimmune disease affecting approximately 1% of the global population, which is characterised by synovitis, cartilage damage, and bone resorption in the joint [1]. Moreover, RA is associated with fatigue [2], cervical spine disease, carpal tunnel syndrome [3], interstitial lung disease [4], cardiovascular disease [5], depression [6], and sleep disorders [7]. RA can cause personal and emotional problems, and impose a significant socio-economic burden [8,9]. The medical treatment of RA includes biological disease-modifying anti-rheumatic drugs, conventional disease-modifying anti-rheumatic drugs, and analgesics. However, these available therapies cannot treat the disease completely and exert significant side effects. Therefore, the development of new therapeutics for RA is needed [10,11].

RA is characterised by synovitis accompanied by the infiltration of immune cells [12], including T cells, B cells, dendritic cells [13], neutrophils [14], and macrophages [15]. Studies have indicated that anti-citrullinated protein antibodies (ACPAs) and inflammatory cytokines, such as interleukin (IL)-1β, IL-6, IL-17, and tumour necrosis factor-alpha (TNF-α), are pivotal mediators in RA [16]. ACPAs are also a specific diagnostic biomarker for RA [17]. Furthermore, oxidative stress caused by reactive oxygen species (ROS) is crucial in joint inflammation, and RA patients exhibit high level of ROS in serum [18,19]. Hence, antioxidant drugs may be effective in treating RA [20]. Collagen-induced arthritis (CIA) in mice recapitulates the clinical and pathogenic characteristics of human RA, and they are widely used to study RA [21].

Kan-Lu-Hsiao-Tu-Tan (KLHTT), a Chinese medicine (CM), has been used to treat inflammatory conditions such as RA, systemic lupus erythematosus, dermatomyositis [22], sinusitis, gingivitis, gastritis, hepatitis [23], and dermatitis [24]. Our previous study demonstrated that KLHTT exerts ROS scavenging ability and anti-inflammatory activity in human neutrophils and exhibits anti-psoriatic activity in mice [25]. However, the pharmacologic effects of KLHTT on RA, another significant autoimmune inflammatory disorder, have not yet been elucidated. Herein, in this study, we aimed to investigate the anti-arthritic effects of KLHTT in CIA mice and evaluate its value in the treatment of RA.

## 2. Materials and Methods

### 2.1. Reagents

KLHTT (batch number: 0503-2-403-01) was supplied by Sun Ten Pharmaceutical Corporation, New Taipei City, Taiwan. Chicken collagen type II (CII) was purchased from Chondrex, Inc., Woodinville, WA, USA. Mycobacterium tuberculosis H37RA was bought from Difco Laboratories Inc., Detroit, MI, USA. Methotrexate (MTX) and EDTA were ordered from Bio Basic Inc., Toronto, ON, Canada. Enzyme-linked immunosorbent assay (ELISA) kits (for IL-1β, IL-6, and TNF-α) were obtained from eBioscience, San Diego, CA, USA. An IL-17A ELISA kit was purchased from R&D Systems Inc., Minneapolis, MN, USA. A hydrogen peroxide assay kit was purchased from Cell Biolabs, Inc., San Diego, CA, USA. Goat anti-mouse IgG1 and goat anti-mouse IgG2a secondary antibodies were purchased from Jackson ImmunoResearch Laboratories, West Grove, PA, USA. 2,2’-Azinobis [3-ethylbenzothiazoline-6-sulfonic acid]-diammonium salt (ABTS) substrate solution was ordered form Roche Diagnostic Systems, South San Francisco, CA, USA. A ^3^H labelled thymidine was purchased from Amersham Pharmacia Biotech, Arlington Heights, IL, USA. Brefeldin A and Freund’s adjuvant were bought from Sigma-Aldrich, St. Louis, MO, USA. Phycoerythrin (PE)-conjugated anti-mouse CD4 (clone GK1.5), FITC-conjugated anti-mouse IL-17A (clone TC11-18H10.1), and FITC-conjugated anti-mouse interferon (IFN)-γ (clone XMG1.2) antibodies were ordered from Biolegend, San Diego, CA, USA. Formalin was bought from AVANTOR, Center Valley, PA. Bovine serum albumin (BSA) was purchased from EMD Millipore, Billerica, MA, USA. Tween 20 was obtained from EMD Millipore, Alsace, France.

### 2.2. KLHTT Preparation

The herbs of KLHTT were purchased and identified by Sun Ten Pharmaceutical Corporation, New Taipei City, Taiwan. A total of 27.93 g of herbs (6.25 g Soapstone, 4.58 g *Artemisia capillaris* Thunb. (seedling), 4.17 g *Scutellaria baicalensis* Georgi (root), 2.50 g *Acorus gramineus* Soland. (rhizome), 2.08 g *Clematis armandii* Franch. (rattan and stem), 2.08 g *Fritillaria cirrhosa* D. Don (bulb), 1.67 g *Pogostemon cablin* (Blanco) Benth. (plant shoot), 1.67 g *Forsythia suspensa* (Thunb.) Vahl (fruits), 1.67 g *Amomum kravanh* Pierre ex Gagnep. (fruits), 1.67 g *Mentha haplocalyx* Briq. (stem and leaf plot), and 1.67 g *Belamcanda chinensis* (L.) DC (rhizome)) was extracted by boiling water (12 times the weight of the herbs) for 1 h, and then concentrated to a voucher specimen (CGU_KLHTT-01) by the freeze dryer (LABCONCO, Kansas City, MO, USA) [25]. The voucher specimen complied with Chang Gung University guidelines.

### 2.3. Ultra-Performance Liquid Chromatography-Tandem Mass Spectrometry

The chemical profile of KLHTT extract was obtained using ultra-performance liquid chromatography-tandem mass spectrometry (UPLC-MS/MS) comprising an LC-30AD pump, SIL-30AC auto-sampler, CTO-20AC column, and SPD-M20A photodiode array detector (Nexera X2, Shimazu, Kyoto, Japan). Prior to being loaded onto the UPLC column, 1 mg of KLHTT extract was first dissolved in 1 mL of methanol and filtered through a 0.45 μm membrane. Sample injections of 1 μL were then performed automatically. Liquid chromatography was performed using a CORTECS UPLC C18 column (90Å, 1.6 µm, 2.1 mm × 100 mm) (Waters, Milford, MA, USA). The mobile phase was a mixture of MeCN (A) and water (W, containing 0.1% formic acid). A gradient sequence was executed as follows: 0–10 min, 10–20% A; 10–14 min, 20–25% A; 14–24 min, 25–30% A; 24–28 min, 30–40% A; 28–33 min, 40–50% A; 33–38 min, 50–75% A; 38–40 min, 75–100% A; and 40–43 min, 100% A. The column temperature was set at 35 °C. The flow rate was at 0.4 mL/min. The range of detection wavelengths was fixed in the 190–500 nm.

Multiple reaction monitoring (MRM) experiments (in negative) were carried out using Shimazu LCMS-8045 triple quadrupole mass spectrometry to identify the constituents of KLHTT extract. The precursor ion settings of the corresponding profiling peaks were determined using the full scan experiment (50–1000 amu). The product ions were settled according to previously reported data. The dwell time was fixed at 100 ms and the collision energy was set at 25–45 eV. All MS data were acquired and processed using LCMS LabSolutions software Version 5.93 (Shimazu, Kyoto, Japan).

### 2.4. Experimental Animals

DBA/1J mice (male, six- to eight-week old, weight 20–22 g) were purchased from Jackson Laboratories (Bar Harbor, ME, USA) and maintained at 20–25 °C with half day light/dark cycle under a specific pathogen-free condition. All mice were treated according to the guidelines of the Institutional Animal Care and Use Committee of National Chung Hsing University (NCHU). The study protocol was approved by NCHU ethics committee (approval code: 109-115).

### 2.5. CIA Model Establishment

CIA was induced by active immunisation with chicken CII [26]. Briefly, 2 mg/mL CII was dissolved in 10 mM acetic acid solution and emulsified with an equal volume of complete Freund’s adjuvant containing Mycobacterium tuberculosis H37RA (250 μg/mouse). The mixture (200 μL/mouse) was intradermally injected at the base of the tail. Incomplete Freund’s adjuvant and CII were administered as booster injections to the mice on day 21 after the first immunisation. ddH_2_O, KLHTT, or MTX was administered orally once a day from day 21 to 42. Mice were divided into four groups (*n* = 6/group) randomly as follows: Group I, Normal; Group II, Vehicle (ddH_2_O) + CII; Group III, KLHTT (50 mg/kg) + CII; Group IV, KLHTT (100 mg/kg) + CII. MTX (0.5 mg/kg) was used as a positive control. Mice were euthanized with CO_2_ exposure (100% CO_2_ for 5 min) by experienced experimenters humanely on day 42. The arthritis severity score was recorded every 3 days after the treatment of drugs. The biochemical and histological assays were performed on day 42.

### 2.6. Assessment of Clinical Arthritis Severity

The body weight and arthritis severity score were obtained [26]. The arthritis severity score was evaluated as: 0, no swelling nor redness; 1, mild swelling and redness restricted to the tarsals or the ankle joint; 2, mild swelling and redness from the tarsals to the ankle; 3, moderate swelling and redness extending to the metatarsal joints; 4. severe swelling and redness from the ankle to the foot and the digits, or limb ankyloses. In addition, paw volume was measured using a plethysmometer 37,140 (Ugo Basile SRL, Comerio, VA, Italy).

### 2.7. Assessment of Histological Arthritis Severity

After the mice were humanely sacrificed, the hind limbs were fixed in 10% buffered formalin, decalcified in 15% EDTA, and embedded in paraffin. Serial paraffin sections (5 μm) were stained with haematoxylin and eosin. The severity of histopathological lesions was scored [26] as follows: 0, normal appearance; 1, mild infiltration of inflammatory cells, mild pannus front, and minimal cartilage damage; 2, moderate infiltration of inflammatory cells, erosive pannus front, and moderate cartilage damage; 3, diffuse infiltration of inflammatory cells, severe cartilage damage and bone resorption.

### 2.8. Measurement of Pro-Inflammatory Cytokine Levels

Hind paw was dissected and homogenised in ice-cold saline using a tissue homogeniser. After being centrifuged at 3000 rpm (4 °C, 10 min, twice), the hind paw homogenates were harvested. Blood was collected from the heart. The levels of cytokines in hind paw homogenates and serum were measured by ELISA [16].

### 2.9. Measurement of the Concentrations of Oxidative Markers

Malondialdehyde (MDA) concentration was determined by thiobarbituric acid reactive substances assay at 532 nm. The standard curve was obtained using 1,1,3,3-tetramethoxypropane. Hydrogen peroxide (H_2_O_2_) concentration was measured using a colorimetric OxiSelect™ hydrogen peroxide assay kit at 560 nm [16].

### 2.10. Anti-Collagen Type II Antibody Analysis

Serum samples were diluted 1:250 for IgG1 or 1:125 for IgG2a in Tris-buffered saline (1% BSA and 0.5% Tween 20, pH 8.0), and then transferred to CII (10 μg/mL) pre-coated 96-well plates (Microtiter^TM^, Thermo Fisher Scientific, Roskilde, Denmark) at 4 °C overnight. The plates were washed and incubated with goat anti-mouse secondary antibodies IgG1 (1:500 dilution) or IgG2a (1:500 dilution) at 25–27 °C for 1 h. After being washed, ABTS substrate was added and the reactions were stopped by adding H_2_SO_4_. The level of IgG1 and IgG2a was measured at 450 nm by an ELISA reader (Sunrise, Tecan Inc., Männedorf, Switzerland) [16].

### 2.11. Splenocyte Proliferation Assay

Splenocytes (4 × 10^5^ cells/well) were cultured with chicken CII (50 μg/mL) at 37 °C for 40 h, and then incubated with ^3^H for 8 h. Cell proliferation was evaluated by radioactive thymidine incorporation [16].

### 2.12. Intracellular Staining

Splenocytes (1 × 10^6^ cells/well) were cultured with chicken CII (50 μg/mL) at 37 °C for 48 h, and then brefeldin A (5 μg/mL) was added for 6 h. Cells were harvested and extracellularly stained with PE-conjugated anti-mouse CD4 antibodies. After being fixed and permeabilised with Cytofix/Cytoperm solution (BD Pharmingen), cells were then intracellularly labelled with FITC-conjugated anti-mouse IL-17A and anti-mouse IFN-γ antibodies. Splenocytes were detected by an Accuri C5 flow cytometer (Accuri Cytometers, Ann Arbor, MI, USA) and analysed by BD Accuri™ C6 Plus software [26].

### 2.13. Statistical Analysis

Data are presented as mean ± SD. Statistical analyses were performed using one- or two-way ANOVA followed by Tukey’s honest significant difference test. A *p*-value < 0.05 was considered statistically significant.

## 3. Results

### 3.1. Identification of Flavone Derivatives in KLHTT Extract

**Figure 1:**
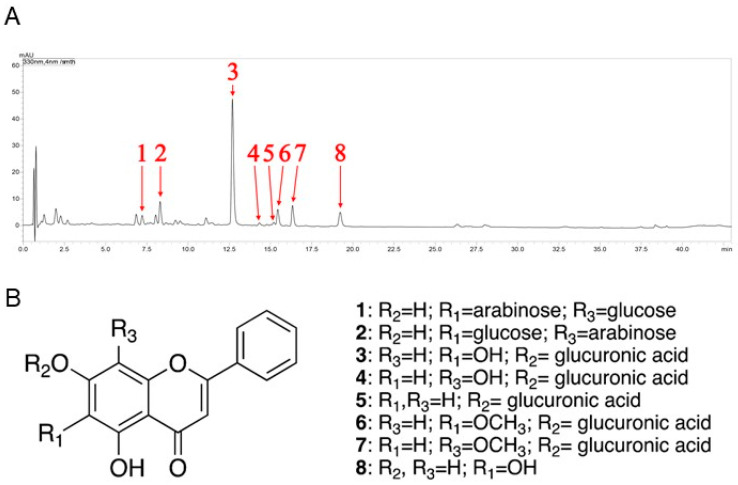
The chemical fingerprint of KLHTT. (**A**) Ultra-performance liquid chromatography with photodiode array detector chromatogram (λ = 330 nm) of KLHTT extract. (**B**) The flavonoid derivatives in KLHTT extract were identified by comparing specific liquid chromatography-tandem mass spectrometry monitoring fragmentations with previously reported data, and were determined to be: chrysin 6-*C*-arabinoside-8-*C*-glucoside (1), chrysin 6-*C*-glucoside-8-*C*-arabinoside (2), baicalin (3), norwogonin-7-*O*-*β*-d-glucuronide (4), chrysin 7-*O*-*β*-d-glucuronide (5), oroxylin A 7-*O*-*β*-d-glucuronide (6), wogonoside (7), and baicalein (8). KLHTT, Kan-Lu-Hsiao-Tu-Tan.

In this study, qualitative analysis of flavonoid-derived constituents was performed by UPLC-MS/MS under 330 nm. The most significant components of the KLHTT extract were flavonoid derivatives (Figure 1A). It has been reported that flavone glycosides are the major constituents of the KLHTT aqueous extract [27]. Moreover, some of the flavonoids and their glycosides such as baicalin and baicalein were found to display significant anti-inflammatory properties [25]. Therefore, we conducted tandem mass (MS/MS) spectrometry experiments to identify the constituents of KLHTT for flavonoids and their glycosides specifically. The specific mass fragmentations were compared with previous references [28,29,30,31,32,33,34], and eight flavonoids were identified: chrysin 6-*C*-arabinoside-8-*C*-glucoside (1), chrysin 6-*C*-glucoside-8-*C*-arabinoside (2), baicalin (3), norwogonin-7-*O*-*β*-d-glucuronide (4), chrysin 7-*O*-*β*-d-glucuronide (5), oroxylin A 7-*O*-*β*-d-glucuronide (6), wogonoside (7), and baicalein (8) (Figure 1B) (Table 1).

### 3.2. KLHTT Exerts Anti-Arthritic Effects in CIA Mice

**Figure 2:**
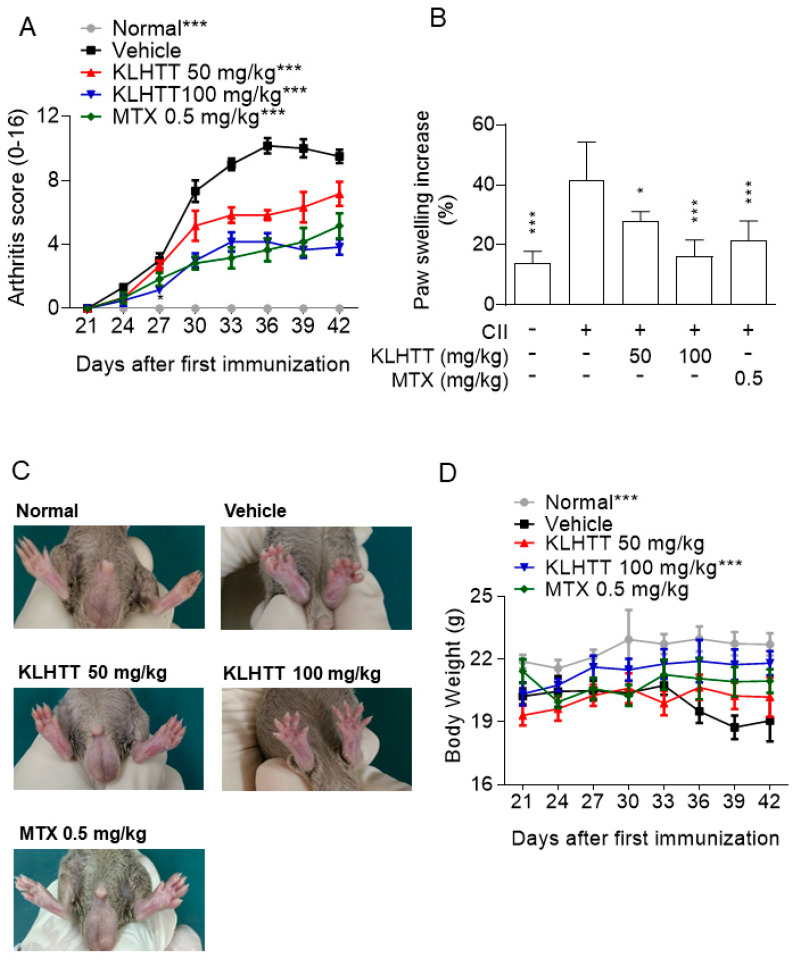
KLHTT ameliorates CIA severity. CIA was induced by active immunisation with chicken CII in DBA/1J mice. Drugs were administered orally once a day from day 21 to 42. (**A**) Arthritis score was monitored every 3 days after the treatment of drugs. Data are expressed as mean ± SD (*n* = 6). *** *p* < 0.001 versus vehicle-treated CIA control mice; two-way ANOVA. (**B**) Paw swelling was assessed using a plethysmometer on day 42. Data are expressed as mean ± SD (*n* = 6). * *p* < 0.05 and *** *p* < 0.001 versus vehicle-treated CIA control mice; one-way ANOVA. (**C**) Representative pictures of hind paws on day 42 are showed. (**D**) Body weight was monitored after the booster immunisation. *** *p* < 0.001 versus vehicle-treated CIA control mice; two-way ANOVA. CIA, collagen-induced arthritis; CII, collagen type II; KLHTT, Kan-Lu-Hsiao-Tu-Tan; MTX, methotrexate.

The CIA mouse model is a well-established and commonly used model mimicking the clinical symptoms and immunopathogenesis of human RA [35]. Immunisation of mice with CII induced increases in clinical arthritis scores, paw volume, and histopathological damage. The normal group exhibited no gross or histological changes. KLHTT (50 and 100 mg/kg) showed inhibitory effects on arthritis severity (Figure 2A) and paw erythema and swelling (Figure 2B,C). The body weight loss in CIA mice was also restored by KLHTT (Figure 2D).

**Figure 3:**
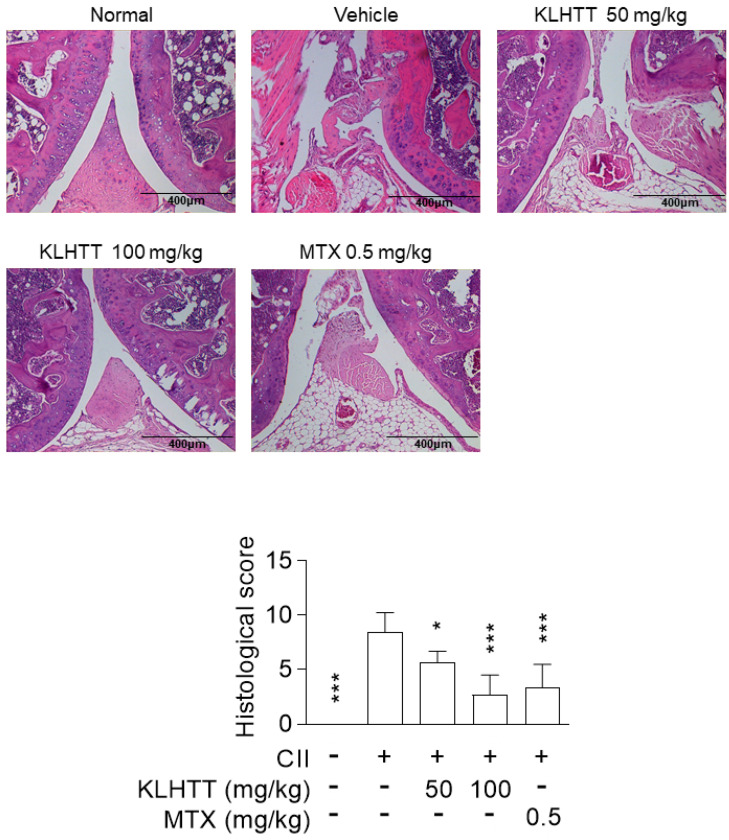
KLHTT reduces joint damage in CIA mice. CIA was induced by active immunisation with chicken CII in DBA/1J mice. Drugs were administered orally once a day from day 21 to 42. Haematoxylin and eosin-stained joint sections from mice of different groups were prepared and pathogenic scores were determined. Original magnification 100×. Bar = 400 μm. Data are expressed as mean ± SD (*n* = 6). * *p* < 0.05 and *** *p* < 0.001 versus vehicle-treated CIA control mice; one-way ANOVA. CIA, collagen-induced arthritis; CII, collagen type II; KLHTT, Kan-Lu-Hsiao-Tu-Tan; MTX, methotrexate.

Histopathological analysis of the CIA mice revealed inflammatory cell infiltration into articular tissues, exudates within the synovial space, synovial hyperplasia, and cartilage erosion. KLHTT-treated mice demonstrated well-preserved joint spaces with minimal inflammatory exudates, normal cartilage structure, and clear synovial spaces, along with improved histological arthritis severity scores (Figure 3). MTX (0.5 mg/kg) was used as a positive control and showed comparable inhibitory effects with KLHTT in CIA mice (Figure 2 and Figure 3).

### 3.3. KLHTT Inhibits Cytokine Production in CIA Mice

**Figure 4:**
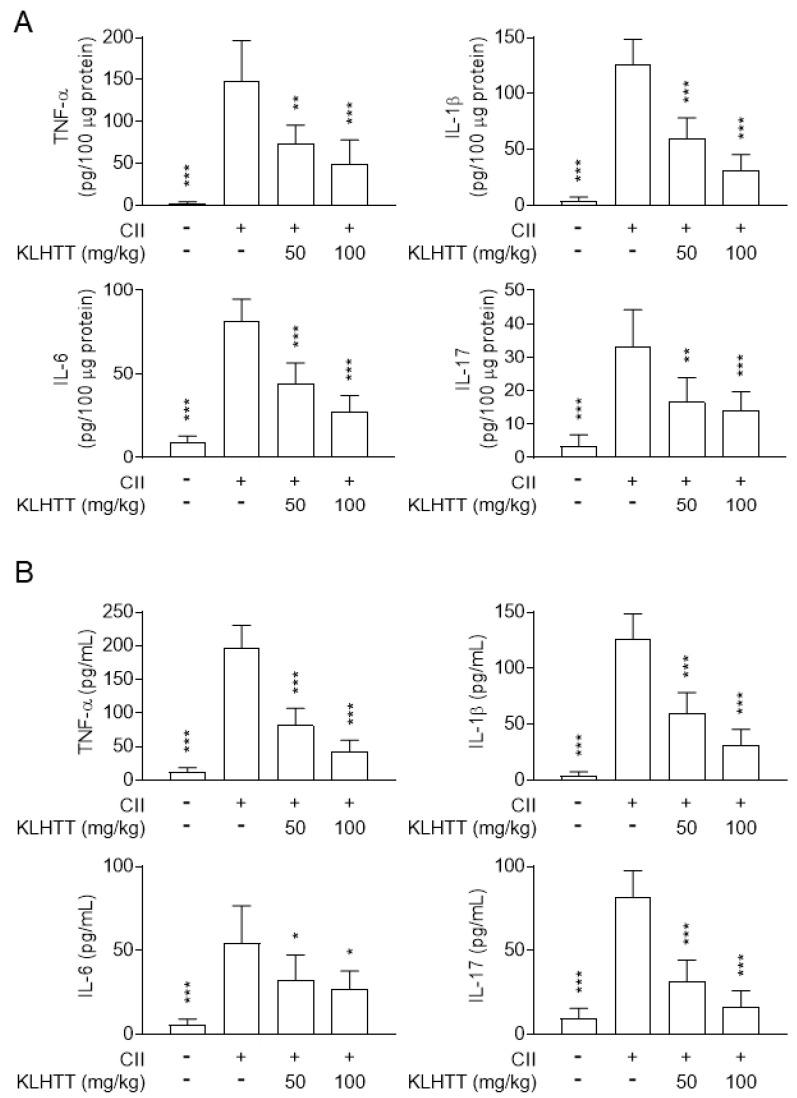
KLHTT inhibits pro-inflammatory cytokine production in CIA mice. CIA was induced by active immunisation with chicken CII in DBA/1J mice. Drugs were administered orally once a day from day 21 to 42. The levels of cytokines in hind paw homogenates (**A**) and serum (**B**) from CIA mice were measured on day 42 by enzyme-linked immunosorbent assay. Data are expressed as mean ± SD (*n* = 6). * *p* < 0.05, ** *p* < 0.01, and *** *p* < 0.001 versus vehicle-treated CIA control mice; one-way ANOVA. CIA, collagen-induced arthritis; CII, collagen type II; IL, interleukin; KLHTT, Kan-Lu-Hsiao-Tu-Tan; TNF-α, tumour necrosis factor-α.

The pathogenesis of RA involves activated T cells promoting macrophages to release pro-inflammatory cytokines [35]. Therefore, the levels of TNF-α, IL-1β, IL-6, and IL-17 in hind paw homogenates and serum samples were measured by sandwich ELISA. KLHTT (50 and 100 mg/kg) treatment inhibited the levels of IL-1β, IL-6, IL-17, and TNF-α in paw homogenates (Figure 4A) and serum samples (Figure 4B) in CIA mice. These results indicated that KLHTT effectively attenuates inflammation in CIA mice.

### 3.4. KLHTT Reduces Oxidative Stress in CIA Mice

**Figure 5:**
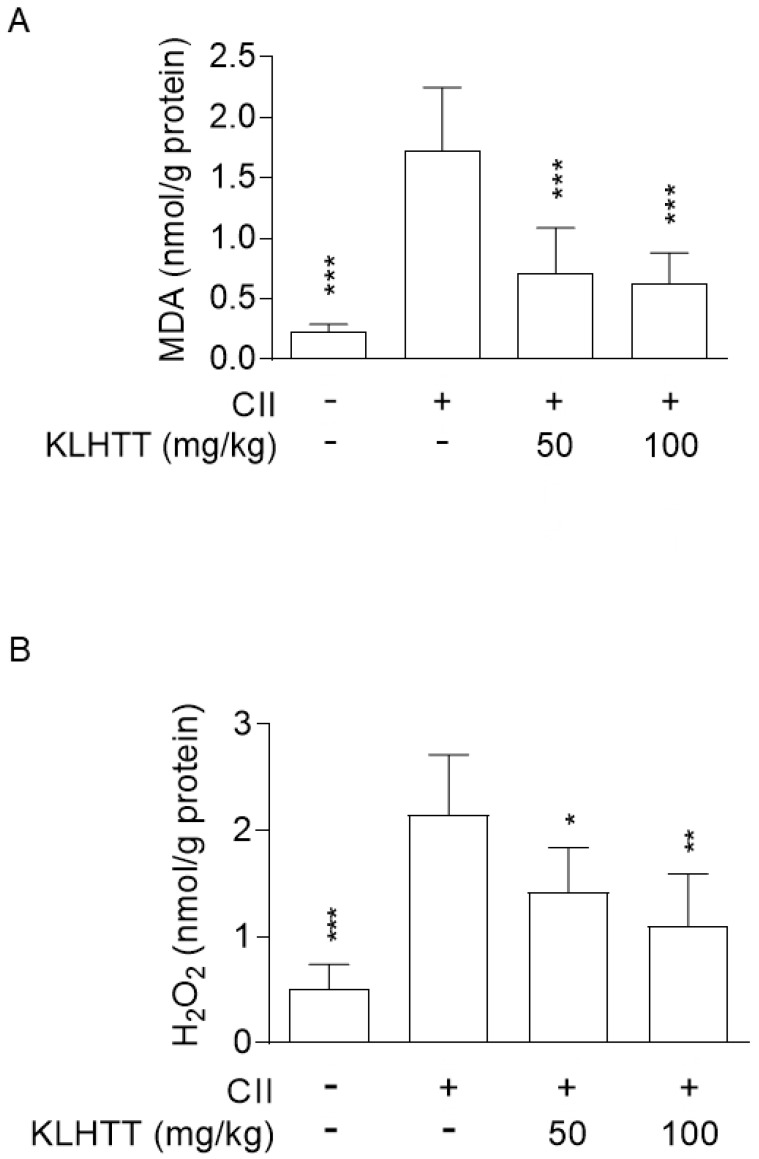
KLHTT reduces the levels of MDA and H_2_O_2_ in CIA mice. CIA was induced by active immunisation with chicken CII in DBA/1J mice. Drugs were administered orally once a day from day 21 to 42. (**A**) MDA (a lipid peroxidation marker) and (**B**) H_2_O_2_ (an ROS marker) were determined on day 42 by the thiobarbituric acid reactive substances assay and the hydrogen peroxide assay kit, respectively. Data are expressed as mean ± SD (*n* = 6). * *p* < 0.05, ** *p* < 0.01, and *** *p* < 0.001 versus vehicle-treated CIA control mice; one-way ANOVA. CIA, collagen-induced arthritis; CII, collagen type II; H_2_O_2_, hydrogen peroxide; KLHTT, Kan-Lu-Hsiao-Tu-Tan; MDA, malondialdehyde; ROS, reactive oxygen species.

RA patients exhibit high level of oxidative stress, which correlates with joint inflammation and may contribute to the chronicity of RA [36]. Significantly elevated levels of MDA and H_2_O_2_ were noted in the hind paw homogenates of CIA mice. KLHTT (50 and 100 mg/kg) significantly reduced the levels of MDA (Figure 5A) and H_2_O_2_ (Figure 5B). These data show that KLHTT protects mice from oxidative damage, which may have contributed to the amelioration of CIA.

### 3.5. KLHTT Inhibits CII-Specific Antibody Production and Splenocyte Proliferation in CIA Mice

**Figure 6:**
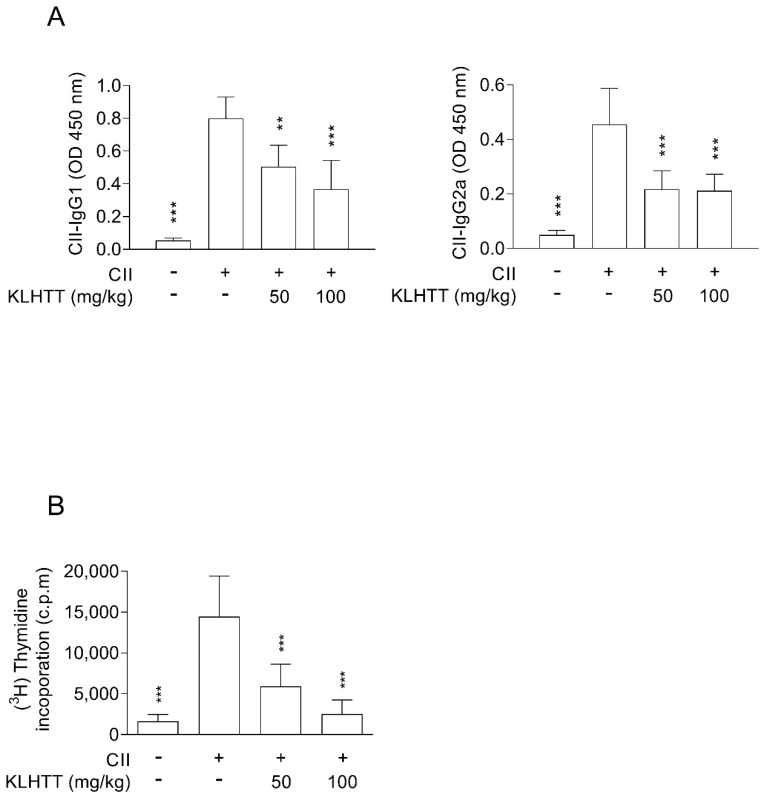
KLHTT inhibits anti-IgG CII antibody production and splenocyte proliferation in CIA mice. CIA was induced by active immunisation with chicken CII in DBA/1J mice. Drugs were administered orally once a day from day 21 to 42. (**A**) The levels of anti-CII IgG1 and IgG2a antibodies were detected on day 42 using enzyme-linked immunosorbent assay. (**B**) Splenocytes were cultured with CII for 40 h, and then cell proliferation was measured by incorporation of (^3^H)-thymidine. Data are expressed as mean ± SD (*n* = 6). ** *p* < 0.01, and *** *p* < 0.001 versus vehicle-treated CIA control mice; one-way ANOVA. CIA, collagen-induced arthritis; CII, collagen type II; KLHTT, Kan-Lu-Hsiao-Tu-Tan.

Autoantibodies targeting IgG play a major role in RA. Similarly, elevated levels of IgG1 and IgG2a antibodies were detected in serum samples from CIA mice. KLHTT significantly suppressed IgG1 and IgG2a antibody production (Figure 6A). Furthermore, KLHTT significantly inhibited the proliferation of CII-induced splenocytes (Figure 6B).

### 3.6. KLHTT Reduces the Levels of Splenic Th1 and Th17 Cells in CIA Mice

**Figure 7:**
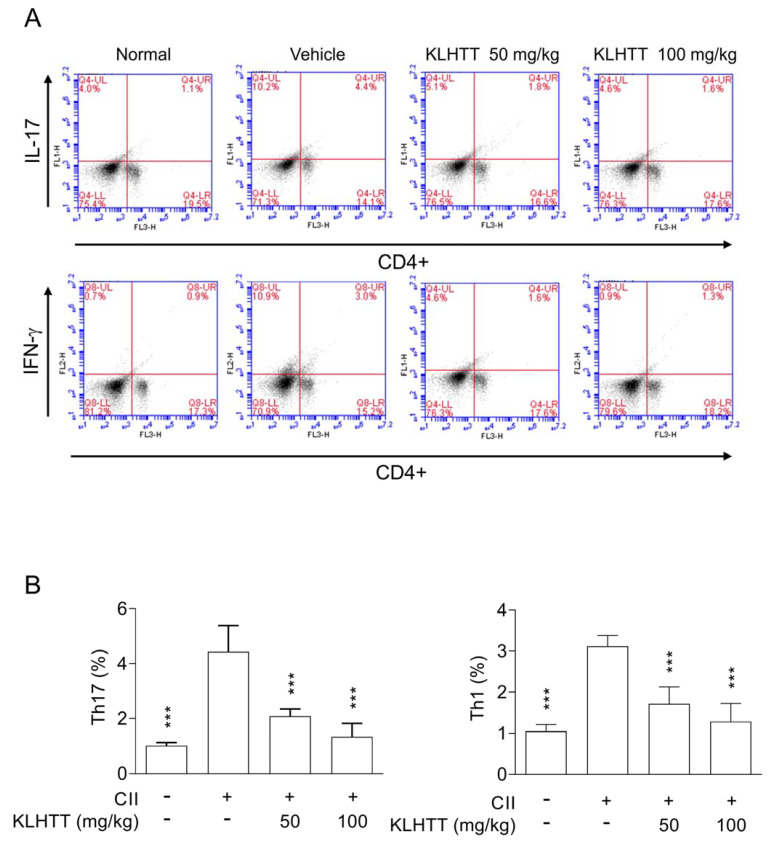
KLHTT reduces the levels of splenic Th1 and Th17 cells in CIA mice. CIA was induced by active immunisation with chicken CII in DBA/1J mice. Drugs were administered orally once a day from day 21 to 42. (**A**) On day 42, splenocytes were cultured with CII for 2 d, and then stained with PE-conjugated anti-CD4 antibodies followed by FITC-conjugated anti-IL-17A or anti-IFN-γ antibodies. Samples were analysed by flow cytometry. (**B**) Bars display the mean ± SD (*n* = 6). *** *p* < 0.001 versus vehicle-treated CIA control mice; one-way ANOVA. CIA, collagen-induced arthritis; CII, collagen type II; FITC, fluorescein isothiocyanate; IL-17, interleukin 17; IFN-γ, interferon gamma; KLHTT, Kan-Lu-Hsiao-Tu-Tan; PE, phycoerythrin.

The pro-inflammatory Th1 and Th17 cell axes play crucial roles in RA. The levels of splenic Th1 and Th17 cells were higher after CII induction. KLHTT significantly decreased the numbers of CD4 ^+^ IFNγ^+^Th1 and CD4^+^IL17A^+^Th17 cells (Figure 7). KLHTT also mitigated the levels of pro-inflammatory cytokines in CIA mice (Figure 4). These results indicated that KLHTT decreases splenic pro-inflammatory Th1 and Th17 cells in CIA mice.

## 4. Discussion

RA is a chronic autoimmune inflammatory disease [1]. Patients with RA have systemic inflammatory comorbidities. The therapeutic armamentarium for RA has expanded from analgesics and nonsteroidal anti-inflammatory drugs to biological disease-modifying anti-rheumatic drugs and conventional disease-modifying anti-rheumatic drugs; however, these available therapies may cause adverse reactions and fail to achieve long-term remission [37]. Therefore, the development of new drugs is required to improve the treatment of RA.

KLHTT is a well-known CM and has been used to treat inflammatory diseases [22]. In this study, we investigated the anti-arthritic effect of KLHTT in CIA mice. Mice actively immunised with CII develop CIA, which closely resembles human RA. CIA mice showed paw erythema and swelling, synovitis, cartilage damage, and bone erosion [35]. KLHTT reduced arthritis severity scores and paw swelling, and restored body weight in CIA mice. KLHTT also decreased inflammatory cell infiltration. Both in CIA and human RA, pro-inflammatory cytokines such as TNF-α, IL-1β, IL-6, and IL-17 trigger autoimmune reactions and enhance chronic inflammation in synovial tissues [38,39]. These pro-inflammatory cytokines activate synovial fibroblasts and chondrocytes to produce enzymes which degrade collagen and proteoglycans, thus damaging adjacent joint tissues [40]. KLHTT significantly reduced the levels of TNF-α, IL-1β, IL-6, and IL-17 in serum samples and joint homogenates. Therefore, KLHTT exerts local and systemic anti-inflammatory effects, which may explain its anti-arthritic activity.

Autoantibodies such as rheumatoid factor and ACPAs can be detected in 50–80% of RA patients. Increased levels of anti-CII IgG correlate with elevated TNF-α and IL-6 in RA patients [41]. In CIA mice, anti-CII antibodies initiate arthritis and CII-reactive T cells promote the progression of the disease [42]. In this study, KLHTT reduced the levels of anti-CII IgG1 and IgG2a in serum samples from CIA mice. Anti-CII IgG2 autoantibodies are the predominant subclass of autoantibodies in CIA mice [35]. Additionally, KLHTT inhibited CII-induced splenocyte proliferation and reduced the levels of splenic Th1 and Th17 cells. These results indicate that KLHTT has immunomodulatory effects in CIA.

Oxidative stress is involved in the pathogenesis of RA [36,43]. In this study, KLHTT significantly decreased the levels of H_2_O_2_ (an ROS marker) and MDA (a lipid peroxidation marker) in joint homogenates from CIA mice. MDA-related reactions are highly immunogenic. MDA levels correlate with RA severity and can be used to predict RA severity [43]. In addition, autoreactive T cells such as Th1 and Th17 cells are crucial in the pathogenesis of RA [44]. The newly diagnosed RA patients have higher levels of serum Th1 and Th17 cells [45]. RA patients show increased Th17 cell infiltration in the synovium [46,47]. Infliximab, an anti-TNF-α antibody, promotes Th1 cell apoptosis in RA patients, thus impeding RA progression [48]. Adalimumab, another anti-TNF-α drug, mitigates the homing of Th17 cells to the synovium, consequently improving joint damage [49]. In this study, KLHTT decreased the levels of splenic Th1 and Th17 cells. The levels of pro-inflammatory cytokines, such as IL-1β, IL-6, IL-17, and TNF-α, were also inhibited by KLHTT. Therefore, we suggest that the regulation of Th1 and Th17 cells is also involved in the anti-arthritic effects of KLHTT.

We identified eight flavonoids from KLHTT, including chrysin 6-*C*-arabinoside-8-*C*-glucoside, chrysin 6-*C*-glucoside-8-*C*-arabinoside, baicalin, norwogonin-7-*O*-*β*-d-glucuronide, chrysin 7-*O*-*β*-d-glucuronide, oroxylin A 7-*O*-*β*-d-glucuronide, wogonoside, and baicalein. Previous studies have reported that baicalin ameliorated CIA in mice by down-regulating Janus kinase 1/signal transducer and activator of transcription 3 signalling and inhibiting IL-17-mediated joint inflammation [50,51]. Baicalein also suppressed human RA fibroblast-like synoviocyte proliferation, induced by IL-1β [52]. Intraperitoneal administration of oroxylin A ameliorated CIA in mice and reduced the levels of IL-1β and IL-6 in human RA fibroblast-like synoviocyte stimulated by TNF-α [53]. Chrysin suppressed nuclear factor-κB and high-mobility group box chromosomal protein in human osteoarthritis chondrocytes stimulated by IL-1β [54,55]. The pathogenesis factors of RA are very complex. A CM formulation that contains various herbs may exhibit synergistic effects [56]. Observably, the anti-arthritic effects of KLHTT were comparable to those of MTX. However, more research is required to prove the synergistic effects of KLHTT in treating RA.

## 5. Conclusions

In summary, our results indicate that KLHTT, a CM formulation, shows significant anti-inflammatory effects, antioxidant activities, and immunomodulatory functions in CIA mice. The present study also demonstrates that KLHTT has potential to treat RA.

## Figures and Tables

**Table 1:** UV and multi-stage mass spectrometry data for the identification of the constituents of Kan-Lu-Hsiao-Tu-Tan extract.

No.	*T*_R_ (Min)	Formula	(–)-ESI-MS/MS Fragment Ions	λ_max_ (Nm)	Identification
1	7.269	C_26_H_28_O_13_	547, 487, 457, 427, 367, 337	271, 317	Chrysin 6-*C*-arabinoside-8-*C*-glucoside
2	8.249	C_26_H_28_O_13_	547, 457, 427, 367, 337	271, 317	Chrysin 6-*C*-glucoside-8-*C*-arabinoside
3	12.674	C_21_H_18_O_11_	445, 269, 251, 223, 197, 113	276, 316	Baicalin
4	14.321	C_21_H_18_O_11_	445, 269, 241, 225, 171	278, 347	Norwogonin-7-*O*-*β*-d-glucuronide
5	15.209	C_21_H_18_O_10_	429, 253, 209, 143, 113	267	Chrysin 7-*O*-*β*-d-glucuronide
6	15.455	C_22_H_20_O_11_	459, 283, 268	271, 311	Oroxylin A 7-*O*-*β*-d-glucuronide
7	16.345	C_22_H_20_O_11_	459, 283, 268	273, 340	Wogonoside
8	19.220	C_15_H_10_O_5_	269, 251, 241, 223, 195, 169, 136	275, 324	Baicalein
